# Synthesis and Biological Evaluation of Analogues of Butyrolactone I as PTP1B Inhibitors

**DOI:** 10.3390/md18110526

**Published:** 2020-10-23

**Authors:** Bihong Hong, Jianlin He, Chaochun Fan, Chao Tang, Qingqing Le, Kaikai Bai, Siwen Niu, Meitian Xiao

**Affiliations:** 1 Third Institute of Oceanography, Ministry of Natural Resources, Xiamen 361005, China; jlhe@tio.org.cn (J.H.); wulixiaotangtang@163.com (C.T.); leqingqing@tio.org.cn (Q.L.); kkbai@tio.org.cn (K.B.); niusiwen@tio.org.cn (S.N.); 2 Technical Innovation Center for Exploitation of Marine Biological Resources, Ministry of Natural Resources, Xiamen 361005, China; fanchaochun1201@163.com; 3 College of Chemical Engineering, Huaqiao University, Xiamen 361021, China

**Keywords:** butenolide, insulin resistance, secondary metabolites, chirality

## Abstract

In recent years, a large number of pharmacologically active compounds containing a butenolide functional group have been isolated from secondary metabolites of marine microorganisms. Butyrolactone I was found to be produced by *Aspergillus terreus* isolated from several marine-derived samples. The hypoglycemic activity of butyrolactone I has aroused our great interest. In this study, we synthesized six racemic butenolide derivatives (namely BL-1–BL-6) by modifying the C-4 side chain of butyrolactone I. Among them, BL-3 and BL-5 improved the insulin resistance of HepG2 cells and did not affect the proliferation of RIN-m5f cell line, which indicated the efficacy and safety of BL-3 and BL-5. Furthermore, BL-3, BL-4, BL-5, and BL-6 displayed a significant protein tyrosine phosphatase 1B (PTP1B) inhibitory effect, while the enantiomers of BL-3 displayed different 50% percentage inhibition concentration (IC_50_) values against PTP1B. The results of molecular docking simulation of the BLs and PTP1B explained the differences of biological consequences observed between the enantiomers of BL-3, which supported BLs as PTP1B inhibitors, and also indicated that the chirality of C-4 might influence the inhibitory effect of the BLs. Our findings provide a novel strategy for the development of butyrolactone derivatives as potential PTP1B inhibitors for the treatment of type 2 diabetes mellitus.

## 1. Introduction

Type 2 diabetes mellitus (T2DM), characterized by high blood sugar in the context of insulin resistance (IR) and a relative lack of insulin [1], is a long-term, non-communicable, multisystem disease that has reached epidemic proportions [2]. Chronic exposure to hyperglycemia affects the microvasculature, eventually leading to diabetic blindness, renal failure, and several neurological disorders with a high impact on the quality of life and overall life expectancy [3]. T2DM is associated with a significant shorter life expectancy [4,5]. Furthermore, accumulating data suggest that younger adults (aged <40 years) with T2DM with a more rapid deterioration of β-cell function than in later-onset T2DM, has increased in both developing and developed countries [6]. Therefore, T2DM and its related complications represent an urgent public health problem.

The living environment is harsh to marine invertebrates such as sea hares, sponges, etc. A large number of epiphytes help them to survive by producing secondary metabolites that may be involved in the chemical defense of the host [7,8]. The metabolites display antibacterial, antiviral, antitumor and other activities [9,10,11]. Butenolide, a collective name for α,β-unsaturated lactones with four carbon heterocyclic rings produced by a variety of marine organisms, is drawing increasing attention in drug discovery [12,13,14,15,16]. Butenolide derivatives display an impressive range of pharmacological properties, including anti-inflammatory [17,18], antibacterial [19], antiviral [20], and antitumor activities [21,22], while aromatic butenolides were found to have α- and β-glucosidase inhibitory activities [23,24]. Protein tyrosine phosphatase 1B (PTP1B) is one of the key members of the PTP family and an important hypoglycemic target which plays a very important role in catalyzing protein tyrosine dephosphorylation and regulating multiple signal transduction pathways [25]. However, the effects of the butenolides on PTP1B were less frequently reported. 

Butyrolactone I is a butenolide that can be produced by several marine-derived *Aspergillus terreus* [26,27,28]. Butyrolactone I has various biological activities. It regulates cell cycle by selectively inhibiting cyclin-dependent kinases (CDKs), including CDK1, CDK2, and CDK5 [29,30]. It is also an efficient inhibitor of the α-glucosidase with a 50% percentage inhibition concentration (IC_50_) of 52.17 μM [31], and has antioxidant activities with an IC_50_ of 51.39 μM [32]. Recently, it was found to improve T2DM with potent TNF-α lowering properties through modulating gut microbiota in db/db mice [33]. The adiponectin production-enhancing activity of butyrolactone I was explained by its dual modulator activities as both a CDK5 inhibitor and a peroxisome proliferator-activated receptor γ partial agonist [34]. Additionally, both natural and synthetic analogues of butyrolactone I exhibited interesting biological activities, including anti-microbial and antitumor effects [35,36,37].

**Figure 1:**
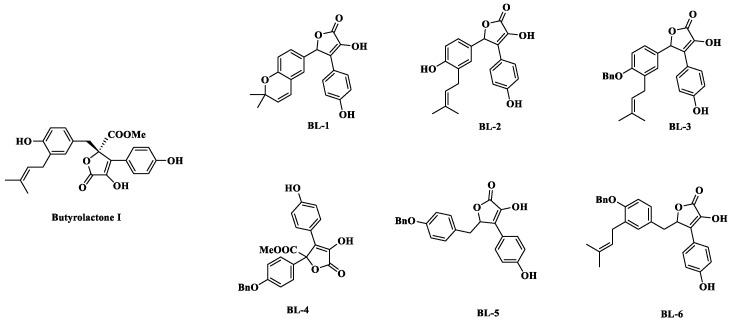
Structures of butyrolactone I and the synthesized compounds BL-1–BL-6.

In this paper, several 2(5H)-furanone compounds (namely BL-1–BL-6) were synthesized by aldol condensation and lactonization based on the modification of the C-4 side chain of butyrolactone I (Figure 1). The hypoglycemic effect of the synthesized compounds was evaluated by PTP1B inhibitory assay, and the effects on glucose uptake was investigated in IR HepG2 cells. Molecular simulation approaches were conducted to explore the interactions between PTP1B and the synthesized compounds. 

## 2. Results and Discussion

### 2.1. Chemistry

**Scheme 1:**
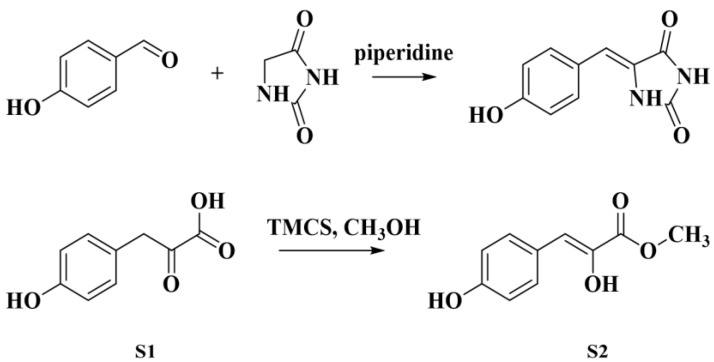
Synthesis of the active methylene intermediate **S2**.

The strategy was to synthesize two intermediates separately and combine them into the lactone ring of the butenolide. Firstly, the active methylene intermediate was synthesized according to Scheme 1. 4-Hydroxybenzaldehyde (**1a**) was condensed with hydantoin (Knoevenagel condensation), and then 4-hydroxyphenylpyruvate (**S1**) was obtained through hydrolysis. Methyl p-hydroxyphenylpyruvate (**S2**) was obtained by quantified esterification of **S1** in methanol under the catalysis of trimethyl chlorosilane (TMCS).

**Scheme 2:**
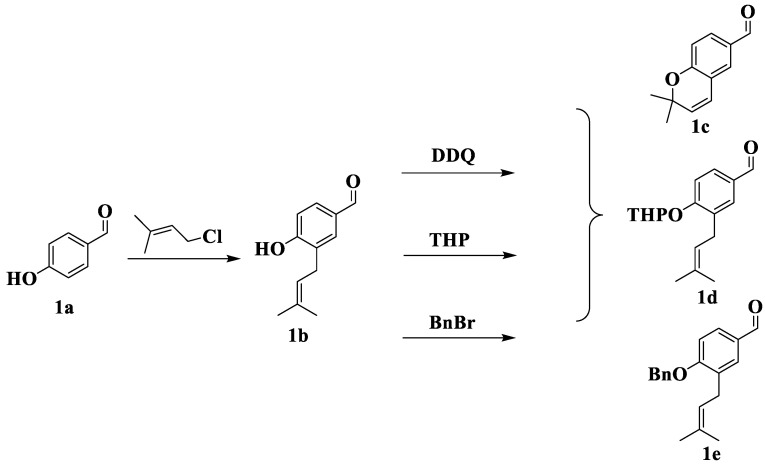
Synthesis of the first type of carbonyl compounds **1c**, **1d** and **1e**.

Secondly, three types of carbonyl compounds were synthesized. Scheme 2 shows the synthesis scheme for the first type. *p*-Hydroxybenzaldehyde (**1a**) generated phenol oxide anions under potassium hydroxide (KOH), which reacted with chloroisoprene to form intermediate **1b** via Friedel–Crafts alkylation [38,39]. Under 2,3-dichloro-5,6-dicyano-1,4-benzoquinone (DDQ), **1b** was refluxed in toluene to form the pyran ring to obtain **1c** [40]. Moreover, **1b** was protected by tetrahydropyran (THP) and benzyl to obtain **1d** and **1e,** respectively [41,42]. 

**Scheme 3:**
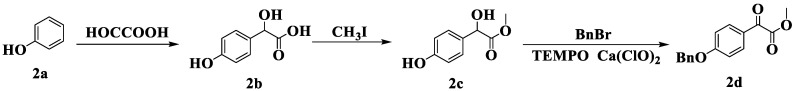
Synthesis of the second type of carbonyl compound **2d**.

The second type was synthesized according to Scheme 3. In the presence of sodium hydroxide (NaOH), 4-hydroxymandelic acid (**2b**) was obtained via alkylation of phenol (**2a**) and glyoxylic acid [43], then esterification with methyl iodide under the catalysis of sodium carbonate to yield 4-hydroxymandelic acid (**2c**) [44]. Compound **2c** underwent 2,2,6,6-tetramethylpiperidine-1-oxyl (TEMPO)/Ca(ClO)₂ oxidation under benzyl protection to produce carbonyl compound **2d** [45]. 

**Scheme 4:**
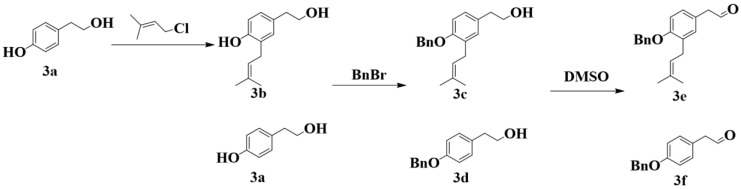
Synthesis of the third type of carbonyl compounds **3e** and **3f**.

The third type was synthesized according to Scheme 4. Phenoxyethanol (**3a**) generated phenol oxide anions under KOH, which reacted with chloroisoprene to form intermediate **3b** via Friedel-Crafts alkylation [38]. Under benzyl protection, **3a** and **3b** were oxidized under dimethyl sulfoxide (DMSO) and sulfur trioxide pyridine complex to obtain **3e** and **3f** via Parikh–Doering oxidation [46]. 

**Scheme 5:**
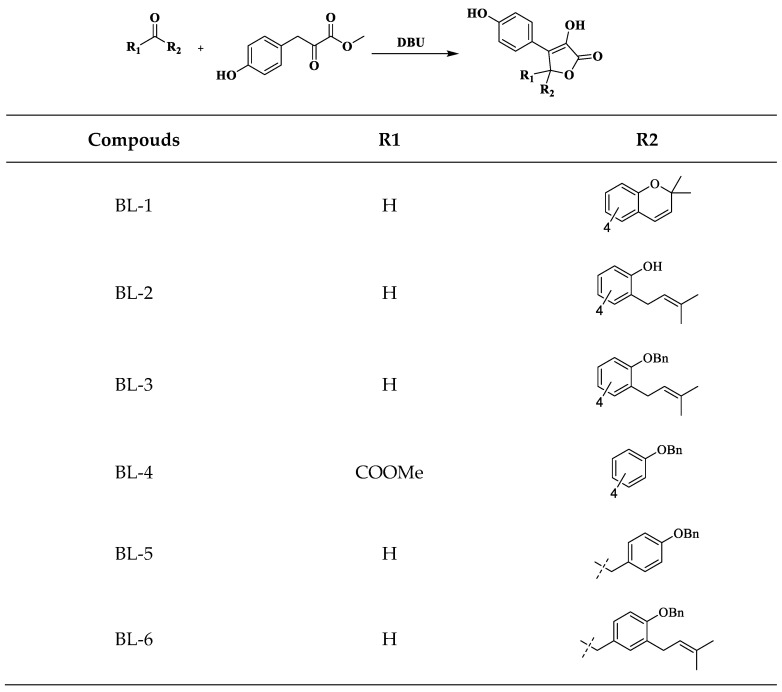
Synthesis of BL-1–BL-6.

Finally, catalyzed by 1,8-diazabicyclo[5.4.0]undec-7-ene (DBU), **1c**, **1d**, **1e**, **2d**, **3e**, and **3f** underwent aldol condensation and lactonization with **S2** respectively (Scheme 5) to obtain butenolides BL-1–BL-6 [47].

Under the catalysis of DBU, C-4 is racemic. We performed chiral resolution and biological activity assay of the preferred compounds (with better biological activities) to evaluate the influence of chirality. The enantiomers of BL-3 and BL-5 were well separated by semi-preparative liquid chromatography with chiral columns.

### 2.2. Biology

The hypoglycemic effect of the synthesized compounds was evaluated by glucose consumed in IR model of HepG2 cell line by high glucose and high insulin stimulation, and rosiglitazone (Ros) was employed as a positive control.

**Figure 2:**
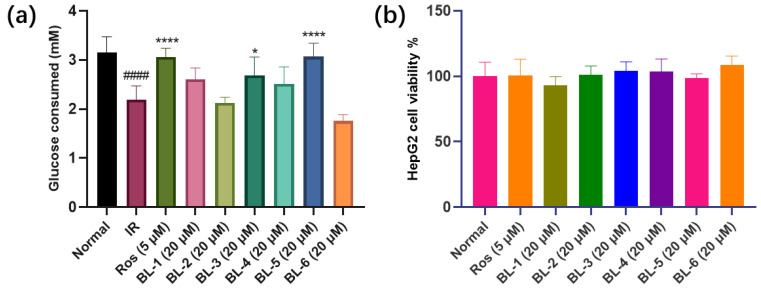
BLs influenced glucose consumed in IR HepG2 cell without inhibiting cell viability at designated concentration. (**a**) Glucose consumed. (**b**) HepG2 cell viability. #### *p* < 0.0001 vs. Normal, * *p* < 0.05 vs. IR, **** *p* < 0.0001 vs. IR. Mean ± SD (*n* = 6).

The results showed that BL-3 and BL-5 displayed significant hypoglycemic effect, and BL-5 at 20 μM had similar potency to Ros at 5 μM (Figure 2a). At the same time, the compounds tested displayed no significant cytotoxicity to HepG2 cells at the designated concentrations (Figure 2b), which is very important to evaluate the hypoglycemic effect since the influence of the cytotoxic effect on the cells has been excluded.

**Figure 3:**
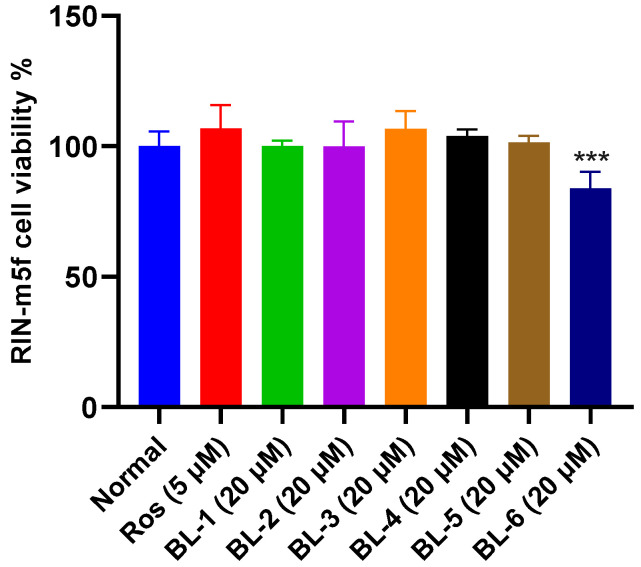
BL-6 inhibited RIN-m5f cell proliferation. *** *p* < 0.001 vs. Normal. Mean ± SD (*n* = 6).

RIN-m5f cell line was used to evaluate the toxicity of the compounds to islet cells. As shown in Figure 3, BL-6 exhibited significant cytotoxicity to RIN-m5f cell line. Additionally, BL-6 did not improve the glucose uptake in IR HepG2 cell (Figure 2a), and therefore BL-6 was not ideal for T2DM treatment.

**Figure 4:**
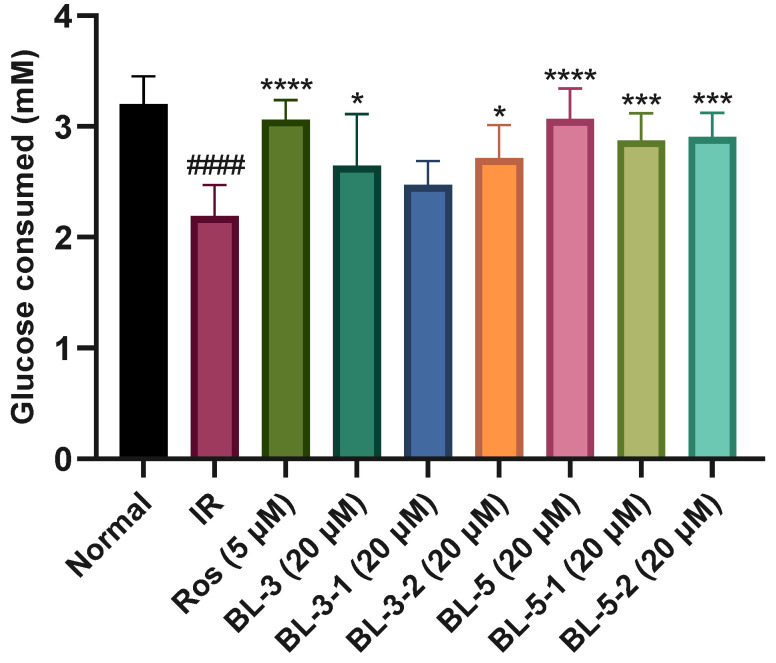
The influence of chirality of BL-3 and BL-5 on glucose consumed. #### *p* < 0.0001 vs. Normal, * *p* < 0.05 vs. IR, *** *p* < 0.001 vs. IR, **** *p* < 0.0001 vs. IR. Mean ± SD (*n* = 6).

Chirality is caused by spatial specific orientation of an asymmetric atom. Although enantiomers have the same physicochemical properties in achiral environments, they may have different biological activities due to their different optical activities. The three-dimensional arrangement of chiral molecules also affects their interaction with enzymes or receptors. The comparison of the hypoglycemic activity of the chiral enantiomers of BL-3 (BL-3-1 and BL-3-2) and BL-5 (BL-5-1 and BL-5-2) is shown in Figure 4. The results indicated that the chiral stereo structure of C-4 has no significant influence on the glucose uptake of BL-5, but might have influence on that of BL-3 (Figure 4).

Based on the results of the IR model, PTP1B inhibitory assay was further established based on reported methods [48] to explain the effects of the synthesized BLs on the glucose uptake.

**Figure 5:**
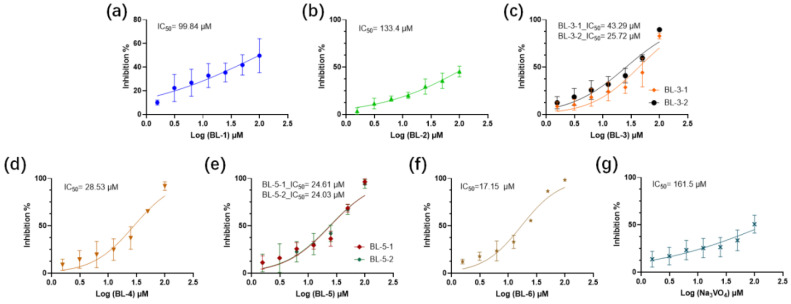
IC_50_ value determinations of (**a**) BL-1, (**b**) BL-2, (**c**) BL-3, (**d**) BL-4, (**e**) BL-5, (**f**) BL-6 and (**g**) Na_3_VO_4_ against PTP1B. Mean ± SD (*n* = 4).

The IC_50_ values were shown in Figure 5. Sodium orthovanadate (Na_3_VO_4_) was used as the positive control. BL-3, BL-4, BL-5, and BL-6 showed strong PTP1B inhibitory activity. BL-3-2 had a significantly lower IC_50_ than BL-3-1, while there were no obvious differences between the IC_50_ values of the enantiomers of BL-5.

**Figure 6:**
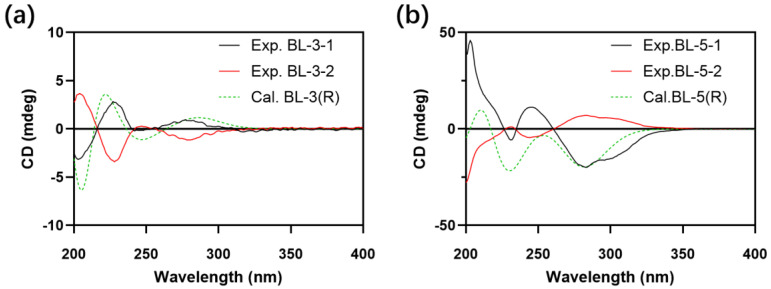
Experimental (Exp.) and calculated (Cal.) Circular dichroism (CD) spectra of (**a**) BL-3 and (**b**) BL-5.

The differences of the amount of glucose consumed and the IC_50_ values against PTP1B were observed between BL-3-1 and BL-3-2, which implied that the three-dimensional arrangement of the C-4 might influence the interactions between PTP1B and the compounds. Therefore, to explore the underlying mechanism, the absolute configuration of C-4 of BL-3 and BL-5 were determined, and molecular docking simulation of the compounds and PTP1B were performed. As shown in Figure 6, BL-3-1 was determined as BL-3(R) and BL-5-1 was determined as BL-5(R).

**Figure 7:**
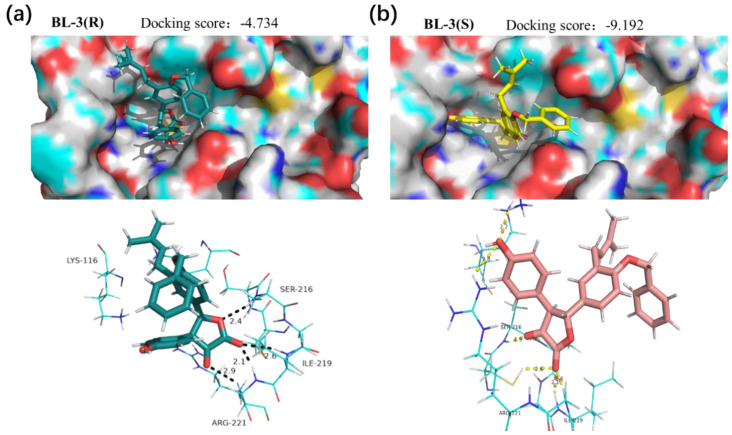
Interaction modes of (**a**) BL-3(R) and (**b**) BL-3(S) with PTP1B. Hydrogen bonds with residues are represented by dashed lines.

**Figure 8:**
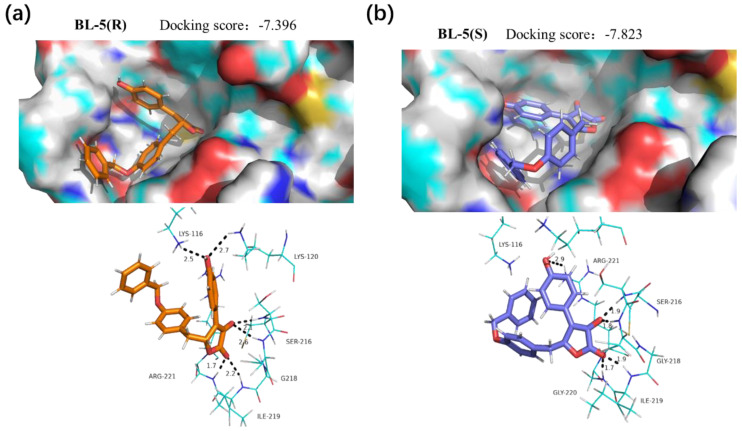
Interaction modes of (**a**) BL-5(R) and (**b**) BL-5(S) with PTP1B. Hydrogen bonds with residues are represented by dashed lines.

The docking data showed that BL-3 and BL-5 could be embedded into the active site of PTP1B, and the lactone core and the 3-phenyl were essential for the docking (Figure 7 and Figure 8). Interestingly, BL-3(R) physically interacted with residues Ser216, Ile219, and Arg221, with a binding energy of –4.73 kcal/mol. While BL-3(S) illustrated more H-bond interactions than BL-3(R), and showed a –9.19 kcal/mol binding energy (Figure 7). The differences of the docking scores indicated that BL-3(S) might have a greater potency of inhibiting PTP1B than BL-3(R), which was supported by the data of PTP1B inhibitory assay shown in Figure 5C. Additionally, as shown in Figure 8, BL-5(R) and BL-5(S) showed close binding energies, indicating close potencies of the enantiomers of BL-5 against PTP1B, which was supported by the data shown in Figure 5E.

Taken together, these simulated molecular docking results were concordant with the results of PTP1B inhibitory assay in vitro (Figure 5), and partly explained the glucose consumed assay data using HepG2 cell line (Figure 4). Therefore, the binding could be responsible for the inhibition of BLs against PTP1B, contributing to the currently observed biological consequences. Our results suggest analogues of butyrolactone I as PTP1B inhibitors. However, the IC_50_ values against PTP1B of the BLs synthesized in this paper were at micromolar level. Therefore, the hypoglycemic effects observed should not attributed to PTP1B inhibition only.

## 3. Materials and Methods

### 3.1. Reagents

Silica gel 60 F254 plates for thin layer chromatography (TLC) and silica gel (300–400 mesh) for column chromatography were purchased from Jiangyou Silica Gel Development, Inc. (Yantai, China). Other reagents and solvents were purchased from Aladdin Reagent Co., Ltd. (Shanghai, China). All moisture- and air-sensitive reactions were carried out using dry solvents under a static atmosphere of nitrogen.

### 3.2. Synthesis Methods

#### 3.2.1. Synthesis of (Z)-2-Hydroxy-3-(4-Hydroxyphenyl) Acrylic Acid (**S1**)

Briefly, 4-Hydroxybenzaldehyde (12.2 g, 0.1 mol, 1.0 eq), hydantoin (11.1 g, 0.11 mol, 1.1 eq) and 20 mL of dry piperidine were mixed and stirred at 130 °C for 50 min. After that, the reaction was cooled to 80 °C, water (400 mL) at 60 °C was added, and stirred for 5 min. Then, the mixture was filtered off. The filtrate was acidified with 20 mL of concentrated hydrochloric acid and stirred at room temperature for 1 h. The resulting precipitate was filtered and washed with ice water (3 × 20 mL). The obtained yellow precipitate was dissolved in 20% NaOH (450 mL), and heated to 145 °C, refluxed for 5 h, then cooled. About 150 mL of concentrated hydrochloric acid was added dropwise at room temperature to adjust the pH to about 2, the mixture was extracted using 150 mL of ether for 3 times, dried over with anhydrous sodium sulfate (Na_2_SO_4_) and concentrated to obtain a yellow solid, which was then recrystallized with 10% dilute hydrochloric acid to obtain about 9.7 g of a light yellow solid in 61% yield.

#### 3.2.2. Synthesis of Methyl (2Z)-2-Hydroxy-3-(4-Hydroxyphenyl) Acrylate (**S2**)

Briefly, 4-Hydroxyphenylpyruvate (**S1**, 0.1 mol) and freshly distilled trimethylchlorosilane (0.2 mol) were dissolved in anhydrous methanol (100 mL). Then, the solution was stirred at 25 °C overnight. After the reaction was completed (monitored by TLC), the reactant was concentrated on a rotary evaporator to obtain the product **S2**, red solid, in 98% yield.

#### 3.2.3. Synthesis of 4-((3-Methylbut-2-en-1-yl) Oxy) Benzaldehyde (**1a**) and 4-Hydroxy-3-(3-Methylbut-2-en-1-yl) Benzaldehyde (**1b**)

*p*-Hydroxybenzaldehyde (12.3 g, 0.1 mol), KOH (5.6 g, 0.1 mol), and pure water (120 mL) were mixed. Then, chloroisopentene (12.5 g, 0.12 mol) was added dropwise with stirring at room temperature. The reaction was monitored with TLC. After the reaction was completed, the pH was adjusted to 3 with 3 mol/L hydrochloric acid, and then the reaction mixture was extracted with ethyl acetate (EA, 3 × 100 mL). The organic phases were combined, washed with saturated sodium carbonate solution and saturated brine, dried over anhydrous Na_2_SO_4_, and concentrated under reduced pressure to obtain a yellow oil, which was then subjected to a silica gel column chromatography with petroleum ether (PE)-EA (8:1, *v/v*) as the mobile phase to obtain 4.12 g of **1a** in 21.7% yield and 3.23 g of **1b** in 17.3% yield.

#### 3.2.4. Synthesis of 2,2-Dimethyl-2H-Chromene-6-Carbaldehyde (**1c**)

To 5 mL of toluene, **1b** (0.39 g, 2 mmol) and DDQ (0.5 g, 2 mmol) was added, and stirred under reflux for 4 h. Then the mixture was filtered off, and the filtrate was concentrated under reduced pressure and purified on a silica gel column with mobile phase of PE-EA (5:1, *v*/*v*) to obtain 0.35 g of a colorless oil (**1c**) in 92.6% yield.

#### 3.2.5. Synthesis of 3-(3-Methylbut-2-en-1-yl)-4-((Tetrahydro-2H-Pyran-2-yl) Oxy) Benzaldehyde (**1d**)

To 5mL of 3,4-dihydro-2H-pyran, **1b** (0.39 g, 2 mmol) and pyridinium p-toluenesulfonate (0.05 g, 0.2 mmol) were added, and then stirred under reflux for 1 h. After the reaction was completed, the solvent was distilled off, and the residue was washed with water (2 × 30 mL) to obtain 0.47 g of colorless oil (**1d**) with a yield of 86.3%.

#### 3.2.6. Synthesis of 4-(Benzyloxy)-3-(3-Methylbut-2-en-1-yl) Benzaldehyde (**1e**)

In a round bottom flask, **1b** (0.39 g, 0.002 mol), K_2_CO_3_ (0.28 g, 0.0021 mol), benzyl bromide (0.38 g, 0.0022 mol), and 20 mL of acetonitrile were mixed, refluxed for 2 h, and filtered off. The filtrate was concentrated under reduced pressure. The residue was then purified on a silica gel column chromatography with PE-EA (10:1, *v*/*v*) as the mobile phase to obtain 0.51 g of a colorless oil (**1e**), with a yield of 91.2%.

#### 3.2.7. Synthesis of 2-Hydroxy-2-(4-Hydroxyphenyl) Acetic Acid (**2b**)

Glyoxylic acid monohydrate (9.2 g, 0.1 mol) and NaOH (4 g, 0.1 mol) were added to 50 mL water to prepare the glyoxylic acid solution. Then, 2 g of NaOH was dissolved in 50 mL water to make a NaOH solution. NaOH (2 g, 0.05 mol), phenol (9.4 g, 0.1 mol), and 50 mL of pure water were added and heated to 50 °C, then the glyoxylic acid solution and the NaOH solution prepared were added dropwise at the same time over a 30 min period, and stirred for another 4 h. After that, the pH was adjusted to 2 with concentrated hydrochloric acid. The mixture was then extracted with EA (3 × 100 mL), dried and concentrated under reduced pressure to obtain a pale-yellow oil, which was recrystallization in PE to obtain 12.7 g of white solid (**2b**) in 76.2% yield.

#### 3.2.8. Synthesis of Methyl 2-Hydroxy-2-(4-Hydroxyphenyl) Acetate (**2c**)

To 80 mL of anhydrous N, N-dimethylformamide (DMF), **2b** (3.4 g, 0.02 mol), methyl iodide (2.9 g, 0.02 mol), and sodium carbonate (2.2 g, 0.021 mol) were added. The resulting mixture was heated to 40 °C, and stirred for about 2 h (monitored with TLC detection). After the reaction, 50 mL of pure water was added, and the pH was adjusted to about 5. The mixture was then extracted with EA (3 × 50 mL), dried, and concentrated under reduced pressure to obtain 2.9 g of white solid (**2c**) in 83% yield.

#### 3.2.9. Synthesis of Methyl 2-(4-(Benzyloxy) Phenyl)-2-Oxoacetate (**2d**)

Briefly, **2c** (1.8 g, 0.01 mol), anhydrous potassium carbonate (2.9 g, 0.011 mol), benzyl bromide (3.8 g, 0.012 mol), and 60 mL of acetonitrile were mixed. The reaction was refluxed at 75 °C, and the progress of the reaction was monitored by TLC. After **2c** was depleted, the reaction mixture was cooled to room temperature, and the catalyst was removed by filtration. TEMPO (0.05 g) and calcium hypochlorite (1.4 g, 0.01 mol) were added to the reaction mixture at 0 °C, and stirred for another 2 h at room temperature, then filtered off, concentrated to obtain the yellow liquid. Silica gel column chromatography (mobile phase of PE-EA, 30:1, *v*/*v*) was employed to obtain pure white solid (**2d**) 1.5 g, in 56% yield.

#### 3.2.10. Synthesis of 4-(2-Hydroxyethyl)-2-(3-Methylbut-2-en-1-yl) Phenol (**3b**)

Phenoxyethanol (**3a**) reacted with chloroisoprene to form **3b** via Friedel–Crafts alkylation. The synthetic process was the same as that of **1b**, described in Section 3.2.3.

#### 3.2.11. Synthesis of 2-(4-(Benzyloxy)-3-(3-Methylbut-2-en-1-yl) Phenyl) Ethan-1-ol (**3c**) and 2-(4-(Benzyloxy) Phenyl) Ethan-1-ol (**3d**)

Briefly, **3a** and **3b** reacted with benzyl bromide to yield **3d** and **3c**, respectively. The synthetic process was the same as that of **1e**, described in 3.2.6.

#### 3.2.12. Synthesis of 2-(4-(Benzyloxy)-3-(3-Methylbut-2-en-1-yl) Phenyl) Acetaldehyde (**3e**) and 2-(4-(Benzyloxy) Phenyl) Acetaldehyde (**3f**)

Sulfur trioxide pyridine complex (3.2 g, 0.02 mol) and DMSO (8 mL) were stirred at room temperature for 15 min to prepare Parikh-Doering intermediate. **3c** (1.5g, 0.0066 mol), 6 mL of triethylamine, 7 mL of DMSO, and 30 mL of dichloromethane were mixed and cool to 0 °C. The prepared intermediate was added dropwise to the reaction solution. After 1 h, the reaction mixture was quenched with 150 mL of ice water, extracted with dichloromethane, washed with saturated brine, dried over anhydrous Na_2_SO_4_, and concentrated under reduced pressure to obtain a colorless oil. Silica gel column chromatography (mobile phase of PE-EA, 8:1, *v*/*v*) was employed to obtain pure white solid (**3e**) 1.05 g, in 70.4% yield. Similarly, **3d** underwent the same reaction process of Parikh-Doering oxidation to yield **3f**.

#### 3.2.13. Synthesis of the 4,5-Diaryl-3-Hydroxy-2(5H) Furanones BL-1–BL-6: General Procedure

At 0 °C, 2-oxo-3-phenylpropionic acid methyl ester (5.27 mmol) and the appropriate carbonyl compound (1.3 equivalents, 6.85 mmol) were added into dry DMF (24 mL). After cooling to 0 °C, DBU (0.80 mL, 5.35 mmol) was added dropwise, the reaction was stirred at 0 °C for 3–5 h, and then poured into hydrogen chloride (HCl, 1mol/L, 50 mL). The organic layer was separated, and the aqueous layer was extracted with EA. The organic layers were combined, dried over Na_2_SO_4_ and concentrated in vacuo, and then purified with column chromatography (mobile phase of EA-PE, 2:1, *v*/*v*) to obtain BL-1–BL-6. The purity of each compound was ≥98.0% by peak area normalization of high-performance liquid chromatography.

#### 3.2.14. Chiral Resolution of BL-3 and BL-5

For BL-3, the chiral resolution was performed on a P230A/P semi-preparative liquid chromatography (Dalian Elite Analytical Instrument Co., Ltd., Dalian, China) with Chiral A4-5 (10 × 250 mm, 5 μm, Column Tek, State College, PA, USA) column to obtain BL-3-1 and BL-3-2. The mobile phase was methanol-water (90:10, *v*/*v*, 0.1% formic acid in water) with isocratic elution. The flow rate was 2.5 mL/min, and the detection wavelength was set at 304 nm.

For BL-5, the chiral resolution was performed on a P230A/P semi-preparative liquid chromatography with Chiral A1-5 (10 × 250 mm, 5 μm, Column Tek) column to obtain BL-5-1 and BL-5-2. The mobile phase was methanol-acetonitrile-water (30:30:40, *v*/*v*, 0.1% formic acid in water) with isocratic elution. The flow rate was 2.5 mL/min, and the detection wavelength was set at 303 nm.

#### 3.2.15. Determination of Absolute Configuration of BL-3 and BL-5

The experimental circular dichroism (CD) spectra were recorded using a Chirascan CD Spectrophotometer (Applied Photophysics Ltd., Surrey, UK).

The conformational search was carried out via random searching in the Sybyl-X 2.0 (Tripos Associates, St Louis, MO, USA) using the MMFF94S force field with an energy cutoff of 3.0 kcal/mol. The conformers were re-optimized with the density functional theory calculations at the b3lyp/6-311+g(2d,p) level in gas phase by the GAUSSIAN 09 program [49]. The energies, oscillator strengths, and rotational strengths of the first 60 electronic excitations were calculated by time-dependent density-functional theory methodology at the b3lyp/6-311++g(2d,p) level in MeOH. The electronic circular dichroism (ECD) spectra were simulated by the overlapping Gaussian function (half the bandwidth at 1/e peak height, σ = 0.3) [50]. To get the final spectra, the simulated spectra of the conformers were averaged according to the Boltzmann distribution theory and their relative Gibbs free energy. The theoretical ECD spectrum of the corresponding enantiomer was obtained direct inversion of the ECD spectrum of the calculated model molecule, respectively.

The absolute configurations of BL-3 and BL-5 were determined by the comparison of their corresponding experimental and calculated CD spectra, respectively.

#### 3.2.16. Nuclear Magnetic Resonance (NMR) Spectroscopy and Mass Spectrometry (MS) for BLs

Briefly, ^1^H-NMR (500 MHz) and ^13^C-NMR (500 MHz) spectra were recorded on a Bruker AC 500 spectrometer (Bruker, Karlsruhe, Germany) in an appropriate solvent with tetramethylsilane (TMS) as an internal reference. High resolution mass spectrometry (HRMS) data were recorded on UPLC/Q-TOF, equipped with an ESI source (Agilent Technologies, Santa Clara, CA, USA).

(BL-1) White solid; yield, 51.2%; ^1^H NMR (500 MHz, DMSO-d6) δ 10.59 (s, 1H, Ar-OH), 9.81 (s, 1H, Ar-C=C-OH), 7.47 (d, *J* = 8.8 Hz, 2H, Ar-H), 7.13 (dd, *J* = 8.3, 2.2 Hz, 1H, Ar-H), 7.02 (d, *J* = 2.2 Hz, 1H, Ar-H), 6.73 (t, *J* = 8.2 Hz, 3H, Ar-H), 6.38 (d, *J* = 9.8 Hz, 1H, Ar-CH=CH), 6.35 (s, 1H, COO-CH-Ar), 5.73 (d, *J* = 9.9 Hz, 1H, CH=CH-C), 1.34 (d, *J* = 2.7 Hz, 6H, CH_3_-C-CH_3_). ^13^C NMR (126 MHz, DMSO-d6) δ 169.79, 158.16, 153.49, 136.96, 131.88, 129.59, 129.52, 129.48, 129.13, 126.03, 122.05, 121.80, 121.58, 116.58, 115.81, 79.87, 76.92, 28.29, 28.27. HRMS (ESI+) calculated for C_21_H_18_O_5_ [M + H]^+^: 351.1227, found: 351.1227.

(BL-2) White solid; yield, 49.4%; ^1^H NMR (500 MHz, DMSO-d6) δ 10.56 (s, 1H, Ar-C=C-OH), 9.79 (s, 1H, Ar-OH), 7.45 (d, *J* = 8.8 Hz, 2H, Ar-H), 7.26 (d, *J* = 8.7 Hz, 2H, Ar-H), 6.91 (d, *J* = 8.7 Hz, 2H, Ar-H), 6.72 (d, *J* = 8.8 Hz, 2H, Ar-H), 6.40 (s, 1H, COO-CH-Ar), 5.40 (m, 1H, CH=C(CH_3_)_2_), 4.49 (d, *J* = 6.7 Hz, 2H, Ar-OCH_2_), 1.73 (s, 3H, CH_3_-C-CH_3_), 1.68 (s, 3H, CH_3_-C-CH_3_). ^13^C NMR (126 MHz, DMSO-d6) δ 169.83, 159.45, 158.13, 137.74, 136.90, 129.64, 129.61, 129.40, 129.09, 122.06, 120.18, 115.77, 115.30, 79.80, 64.75, 25.87, 18.45. HRMS (ESI+) calculated for C_21_H_20_O_5_ [M + H]^+^: 353.1384, found: 353.1378.

(BL-3) White solid; yield, 52.2%; ^1^H NMR (500 MHz, DMSO-d6) δ 10.56 (s, 1H, Ar-OH), 9.80 (s, 1H, Ar-C=C-OH), 7.49 − 7.41 (m, 5H, Ar-H), 7.39 (t, *J* = 7.5 Hz, 2H, Ar-H), 7.33 (d, *J* = 7.3 Hz, 1H, Ar-H), 7.15 (dd, *J* = 8.4, 2.2 Hz, 1H, Ar-H), 7.11 (d, *J* = 2.2 Hz, 1H, Ar-H), 7.01 (d, *J* = 8.5 Hz, 1H, Ar-H), 6.73 (d, *J* = 8.9 Hz, 2H, Ar-H), 6.38 (s, 1H, COO-CH-Ar), 5.19 (m, 1H, CH=C(CH_3_)_2_), 5.08 (s, 2H, Ar-CH2), 3.23 (d, *J* = 7.2 Hz, 2H, Ar-CH_2_-CH), 1.58 (d, *J* = 62.8 Hz, 6H, CH_3_-C-CH_3_). ^13^C NMR (126 MHz, DMSO-d6) δ 169.85, 158.12, 137.53, 136.87, 132.42, 130.28, 129.61, 129.49, 129.30, 129.11, 128.87, 128.24, 127.91, 127.29, 122.56, 122.13, 115.75, 112.43, 79.94, 69.75, 28.90, 25.94, 18.01. HRMS (ESI+) calculated for C_28_H_26_O_5_ [M + H]^+^: 443.1853, found: 443.1859.

(BL-4) Yellow solid; yield, 57.9%; ^1^H NMR (500 MHz, DMSO-d6) δ 11.27 (s, 1H, Ar-OH), 9.91 (s, 1H, Ar-C=C-OH), 7.55 (d, *J* = 8.9 Hz, 2H, Ar-H), 7.45 (d, *J* = 7.2 Hz, 2H, Ar-H), 7.40 (t, *J* = 7.4 Hz, 2H, Ar-H), 7.34 (s, 1H, Ar-H), 7.26 (d, *J* = 8.8 Hz, 2H, Ar-H), 7.04 (d, *J* = 8.9 Hz, 2H, Ar-H), 6.75 (d, *J* = 8.9 Hz, 2H, Ar-H), 5.12 (s, 2H, Ar-CH_2_), 3.74 (s, 3H, COO-CH_3_). ^13^C NMR (126 MHz, DMSO-d6) δ 169.37, 168.25, 159.49, 158.33, 138.89, 137.21, 130.53, 130.36, 128.91, 128.39, 128.23, 121.26, 115.63, 115.18, 87.05, 69.82, 54.01. HRMS (ESI+) calculated for C_25_H_20_O_7_ [M + H]^+^: 433.1282, found: 433.1285.

(BL-5) White solid; yield, 48.5%; ^1^H NMR (500 MHz, Acetone-d6) δ 8.87 (s, 1H, Ar-OH), 8.78 (s, 1H, Ar-C=C-OH), 7.71 (d, *J* = 8.7 Hz, 2H, Ar-H), 7.47 (d, *J* = 7.4 Hz, 2H, Ar-H), 7.39 (t, *J* = 7.4 Hz, 2H, Ar-H), 7.33 (t, *J* = 7.3 Hz, 1H, Ar-H), 7.01 (dd, *J* = 8.8, 2.5 Hz, 4H, Ar-H), 6.88 (d, *J* = 8.6 Hz, 2H, Ar-H), 5.66 (dd, *J* = 6.2, 3.4 Hz, 1H, COO-CH-CH_2_), 5.07 (s, 2H, Ar-CH_2_), 3.33 (dd, *J* = 14.7, 3.3 Hz, 1H, CH-CH_2_-Ar), 2.91 (dd, *J* = 14.7, 6.2 Hz, 1H, CH-CH_2_-Ar). ^13^C NMR (126 MHz, Acetone-d6) δ 157.94, 157.80, 137.57, 136.56, 130.79, 129.26, 129.09, 128.36, 127.74, 127.69, 127.56, 115.71, 114.25, 78.37, 69.48, 38.55. HRMS (ESI+) calculated for C_24_H_20_O_5_ [M + H]^+^: 389.1384, found: 389.1386.

(BL-6) White solid; yield, 43.7%; ^1^H NMR (500 MHz, Acetone-d6) δ 8.86 (s, 1H, Ar-OH), 8.74 (s, 1H, Ar-C=C-OH), 7.70 (d, *J* = 8.8 Hz, 2H, Ar-H), 7.49 (d, *J* = 7.4 Hz, 2H, Ar-H), 7.40 (t, *J* = 7.5 Hz, 2H, Ar-H), 7.33 (d, *J* = 7.3 Hz, 1H, Ar-H), 7.05–6.98 (m, 2H, Ar-H), 6.87 (s, 2H, Ar-H), 6.81 (s, 1H, Ar-H), 5.66 (dd, *J* = 5.7, 3.5 Hz, 1H, COO-CH-CH_2_), 5.22 (m, 1H, CH=C(CH_3_)_2_), 5.08 (s, 2H, Ar-CH_2_), 3.34−3.24 (m, 3H, CH-CH_2_-Ar, Ar-CH_2_-CH), 2.92 (dd, *J* = 14.7, 5.7 Hz, 1H, CH-CH_2_-Ar), 1.69 (s, 3H, CH_3_-C-CH_3_), 1.62 (s, 3H, CH_3_-C-CH_3_). ^13^C NMR (126 MHz, Acetone-d6) δ 168.85, 157.91, 155.31, 137.79, 130.95, 129.42, 129.25, 129.04, 128.34, 128.25, 127.61, 127.40, 127.27, 122.78, 122.51, 115.67, 111.21, 78.27, 69.60, 38.47, 25.06, 17.01. HRMS (ESI+) calculated for C_29_H_28_O_5_ [M + H]^+^: 457.2010, found: 457.2015.

(BL-3-1) White solid; 1H NMR (500 MHz, Acetone-d6) δ 8.82 (s, 2H, Ar-C=C-OH, Ar-OH), 7.58 (d, *J* = 8.8 Hz, 2H, Ar-H), 7.49 (d, *J* = 7.4 Hz, 2H, Ar-H), 7.40 (t, *J* = 7.5 Hz, 2H, Ar-H), 7.34 (d, *J* = 7.3 Hz, 1H, Ar-H), 7.26−7.20 (m, 2H, Ar-H), 7.02 (d, *J* = 8.1 Hz, 1H, Ar-H), 6.82 (d, *J* = 8.8 Hz, 2H, Ar-H), 6.30 (s, 1H, COO-CH-Ar), 5.26 (m, 1H, CH=C(CH3)2), 5.13 (s, 2H, Ar-CH2), 3.33 (d, *J* = 7.3 Hz, 2H, Ar-CH2-CH), 1.68 (s, 3H, CH3-C-CH3), 1.58 (s, 3H, CH3-C-CH3). 13C NMR (126 MHz, Acetone-d6) δ 169.14, 157.79, 157.17, 137.43, 132.12, 130.53, 129.51, 129.29, 128.95, 128.43, 128.37, 127.72, 127.44, 126.96, 122.44, 122.26, 115.28, 111.83, 80.27, 69.71, 24.95, 16.93. HRMS (ESI+) calculated for C_28_H_26_O_5_ [M + Na]^+^: 465.1676, found: 465.1666.

(BL-3-2) White solid; 1H NMR (500 MHz, Acetone-d6) δ 8.91 (s, 2H, Ar-C=C-OH, Ar-OH), 7.58 (d, *J* = 8.8 Hz, 2H, Ar-H), 7.49 (d, *J* = 7.3 Hz, 2H, Ar-H), 7.40 (t, *J* = 7.5 Hz, 2H, Ar-H), 7.33 (t, *J* = 7.3 Hz, 1H, Ar-H), 7.23 (d, *J* = 8.4 Hz, 2H, Ar-H), 7.02 (d, *J* = 8.1 Hz, 1H, Ar-H), 6.82 (d, *J* = 8.8 Hz, 2H, Ar-H), 6.30 (s, 1H, COO-CH-Ar), 5.26 (m, 1H, CH=C(CH3)2), 5.13 (s, 2H, Ar-CH2), 3.33 (d, *J* = 7.3 Hz, 2H, Ar-CH2-CH), 1.68 (s, 3H, CH3-C-CH3), 1.58 (s, 3H, CH3-C-CH3). 13C NMR (126 MHz, Acetone-d6) δ 169.13, 157.79, 157.17, 137.43, 136.72, 132.12, 130.53, 129.52, 129.29, 128.94, 128.38, 127.72, 127.44, 126.97, 122.44, 122.26, 115.29, 111.83, 80.28, 69.71, 24.95, 16.93. HRMS (ESI+) calculated for C_28_H_26_O_5_ [M + Na]^+^: 465.1676, found: 465.1684.

(BL-5-1) White solid; 1H NMR (500 MHz, DMSO-d6) δ 10.01 (s, 2H, Ar-OH, Ar-C=C-OH), 7.60 (d, *J* = 8.5 Hz, 2H, Ar-H), 7.44 (d, *J* = 7.5 Hz, 2H, Ar-H), 7.39 (t, *J* = 7.4 Hz, 2H, Ar-H), 7.33 (t, *J* = 7.2 Hz, 1H, Ar-H), 6.97–6.85 (m, 6H, Ar-H), 5.68 (dd, *J* = 6.1, 3.4 Hz, 1H, COO-CH-CH2), 5.04 (s, 2H, Ar-CH2), 3.21 (dd, *J* = 14.6, 3.0 Hz, 1H, CH-CH2-Ar), 2.76 (dd, *J* = 14.7, 6.3 Hz, 1H, CH-CH2-Ar). 13C NMR (126 MHz, DMSO-d6) δ 158.24, 157.59, 137.62, 131.07, 129.39, 128.84, 128.22, 128.15, 128.11, 122.19, 116.11, 114.69, 78.32, 69.63, 38.78. HRMS (ESI+) calculated for C_24_H_20_O_5_ [M + H]^+^: 389.1384, found: 389.1382.

(BL-5-2) White solid; 1H NMR (500 MHz, DMSO-d6) δ 9.96 (d, 2H, Ar-OH, Ar-C=C-OH), 7.60 (d, *J* = 8.7 Hz, 2H, Ar-H), 7.44 (d, *J* = 7.1 Hz, 2H, Ar-H), 7.39 (t, *J* = 7.5 Hz, 2H, Ar-H), 7.33 (d, *J* = 7.2 Hz, 1H, Ar-H), 6.98–6.84 (m, 6H, Ar-H), 5.68 (dd, *J* = 6.3, 3.4 Hz, 1H, COO-CH-CH2), 5.04 (s, 2H, Ar-CH2), 3.21 (dd, *J* = 14.7, 3.4 Hz, 1H, CH-CH2-Ar), 2.76 (dd, *J* = 14.7, 6.4 Hz, 1H, CH-CH2-Ar). 13C NMR (126 MHz, DMSO-d6) δ 158.23, 157.59, 137.62, 136.85, 131.07, 129.39, 128.84, 128.22, 128.16, 128.11, 122.18, 116.11, 114.69, 78.32, 69.63, 38.77. HRMS (ESI+) calculated for C_24_H_20_O_5_ [M + H]^+^: 389.1384, found: 389.1385.

### 3.3. Biological Evaluation

#### 3.3.1. PTP1B Inhibitory Assay

The tested compounds at designated concentrations were solubilized in DMSO, and 2 μL samples was added to 96-well clear polystyrene plate. The DMSO (2 μL) was set as the full enzyme activity, and Na_3_VO_4_ (2 μL) was set as the positive control. Then, 78 μL of an assay buffer (25 mM Hepes, 150 mM sodium chloride (NaCl), 0.1% bovine serum albumin (BSA), 3 mM dithiothreitol, 1 mM ethylenediaminetetraacetic acid) and 10 μL of the PTP1B (10 μg/mL) was added and incubated for 10 min at 37 °C. The screening was initiated by adding 10 μL *p*-nitrophenyl phosphate (*p*NPP), and the catalysis of *p*NPP (1 mM) was continuously monitored on SpectraMax 340 microplate reader (Molecular Devices, Sunnyvale, CA, USA) at 405 nm for 20 min at 37 °C. The slope of the linear portion of the kinetic curve generated from each well was used to determine the activity of PTP1B. The inhibition effect of the compound was represented by the percentage of the slope of the linear portion of its kinetic curve relative to that of the negative control (DMSO). The IC_50_ was calculated with Prism 8 software (Graphpad, San Diego, CA, USA) from the non-linear curve fitting of the percentage of inhibition (%inhibition) versus the inhibitor concentration [I] by using the following equation: %Inhibition = 100/(1 + [IC_50_/[I]] *^k^*), where *k* is the Hill coefficient.

#### 3.3.2. Establishment of HepG2 Cell Insulin Resistance Model and Evaluation of BLs

HepG2 cells (Cell Bank of Chinese Academy of Sciences, Shanghai, China) were seeded in 96-well plates in normal Dulbecco’s Modified Eagle’ Medium (DMEM, 5.6 mM glucose) supplemented with 10% fetal bovine serum (FBS). The density of the cells in each plate was 1 × 10^4^ cells/mL. The original medium was removed when the cells became adherent. For the control group, the medium was replaced with high glucose DMEM (30 mM glucose). The model of insulin resistance was established by stimulation with high glucose DMEM (30 mM glucose) with 10^−7^ mol/L insulin for 48 h. Then, the cells were treated with drug-free or drug-containing DMEM (5.6 mM glucose) containing 0.2% BSA and milk 1 × 10^−9^ mol/L insulin for 24 h. Glucose consumption was detected with the glucose oxidase method using a glucose assay kit (Cat#BC2500, Solarbio, Beijing, China).

#### 3.3.3. Cell Proliferation Assay

The cytotoxic effect of the synthesized butenolides on RIN-m5f (CRL-11605, American Type Culture Collection, Manassas, VA, USA) and HepG2 cells was determined with a Cell Counting Kit 8 (CCK-8) kit (Dojindo, Tokyo, Japan) [51,52]. Cells were seeded into 96-well plates in DMEM with 10% FBS in a volume of 100 µL per well and cultured overnight at 37 °C. The density of the cells in each plate was 1 × 10^4^ cells/mL. According to the manufacturer’s protocol, after treatment with DMSO or varying concentrations of BLs for 48 h, 10 µL of CCK-8 solution was added to each well and cells were incubated for 2 h at 37 °C. Measure the absorbance at 450 nm of each well on a SpectraMax 340 microplate reader.

#### 3.3.4. Molecular Docking Simulations

Molecular docking was performed using Maestro software (Version 10.2.010, Schrödinger, LLC, New York, NY, USA), and all calculations utilized the standard default settings. The published PTP1B crystal structure (PDB ID: 1NNY) was used as a basis set for this task [53]. The binding region was defined as 15 Å around the ligand 515 in the crystal structure. The types of interaction of the complex and ligand were analyzed by PyMOL Molecular Graphics System (Version 2.32, DeLano Scientific LLC, Palo Alto, CA, USA) after the molecular docking.

## 4. Conclusions

In this study, six racemic butenolides were synthesized by the modification on the C-4 position of butyrolactone I, two of which displayed significant hypoglycemic activities, and the underlying mechanism may be the inhibition of the PTP1B. Additionally, our data suggested that the chirality might influence the interactions between the compounds and PTP1B. Our study provides novel approaches to synthesize butyrolactone derivatives for the treatment of T2DM.

## Abbreviations

BSA	bovine serum albumin
CCK-8	Cell Counting Kit 8
CD	circular dichroism
CDK	cyclin-dependent kinase
DBU	1,8-diazabicyclo[5.4.0]undec-7-ene
DDQ	2,3-dichloro-5,6-dicyano-1,4-benzoquinone
DMEM	Dulbecco’s Modified Eagle’ Medium
DMF	N, N-dimethylformamide
DMSO	dimethyl sulfoxide
EA	ethyl acetate
ECD	electronic circular dichroism
FBS	fetal bovine serum
HRMS	high resolution mass spectrometry
IC_50_ 50%	percentage inhibition concentration
IR	insulin resistant
MS	mass spectrometry
NMR	nuclear magnetic resonance
PE	petroleum ether
*p*NPP	*p*-nitrophenyl phosphate
PTP1B	protein tyrosine phosphatase 1B
Ros	rosiglitazone
T2DM	type 2 diabetes mellitus
TEMPO	2,2,6,6-tetramethylpiperidine-1-oxyl
THP	tetrahydropyran
TLC	thin layer chromatography
TMCS	trimethyl chlorosilane
TMS	tetramethylsilane
